# The effects of amount of home meditation practice in Mindfulness Based Cognitive Therapy on hazard of relapse to depression in the Staying Well after Depression Trial

**DOI:** 10.1016/j.brat.2014.08.015

**Published:** 2014-12

**Authors:** Catherine Crane, Rebecca S. Crane, Catrin Eames, Melanie J.V. Fennell, Sarah Silverton, J. Mark G. Williams, Thorsten Barnhofer

**Affiliations:** a Department of Psychiatry, University of Oxford, Warneford Hospital, Oxford, OX3 7JX, UK; b Centre for Mindfulness Research and Practice, Bangor University, Dean St Building, Bangor, LL57 2PZ, UK

**Keywords:** Mindfulness Based Cognitive Therapy, Depression, Recurrence, Home practice

## Abstract

Few empirical studies have explored the associations between formal and informal mindfulness home practice and outcome in Mindfulness-based Cognitive Therapy (MBCT). In this study ninety-nine participants randomised to MBCT in a multi-centre randomised controlled trial completed self-reported ratings of home practice over 7 treatment weeks. Recurrence of Major Depression was assessed immediately after treatment, and at 3, 6, 9, and 12-months post-treatment. Results identified a significant association between mean daily duration of formal home practice and outcome and additionally indicated that participants who reported that they engaged in formal home practice on at least 3 days a week during the treatment phase were almost half as likely to relapse as those who reported fewer days of formal practice. These associations were independent of the potentially confounding variable of participant-rated treatment plausibility. The current study identified no significant association between informal home practice and outcome, although this may relate to the inherent difficulties in quantifying informal home mindfulness practice. These findings have important implications for clinicians discussing mindfulness-based interventions with their participants, in particular in relation to MBCT, where the amount of participant engagement in home practice appears to have a significant positive impact on outcome.

## Highlights

• We examine the relation between home practice and outcome in people receiving MBCT.
• Formal home practice is negatively associated with hazard of relapse to depression.
• Those who engage in such practice at least three days/week approximately halve hazard of relapse.
• Informal home practice was unrelated to risk of relapse to major depression.
• Effects of formal home practice on outcome are not confounded with treatment plausibility.

## Introduction

Mindfulness Based Cognitive Therapy (MBCT), like other mindfulness-based interventions (MBIs), includes home practice of mindfulness meditations as a core component of the approach. Such practice is regarded as one of the primary vehicles for becoming aware of, and relating differently to, mental habits and thus learning relapse prevention skills (Segal, Williams, & Teasdale, 2013). Thus it would be expected that a significant relationship should exist between amount of home practice completed by people being treated with MBCT and its prophylactic effects on relapse in depression. Despite this, relatively few studies have systematically examined the association between the amount of home practice and outcome in MBCT or other related MBIs. For example, in a recent review of more than 90 empirical studies of MBIs, Vettese, Toneatto, Stea, Nguyen, and Wang (2009) found that only 24 had examined the association between the amount of home practice and subsequent outcome, and only 13 had found at least partial evidence of a positive association. Differences across studies were substantial, and included variability in the nature of the participant group under investigation (clinical/non-clinical), the MBI being studied, how mindfulness practice was assessed (for example by daily diary or retrospective self-report), and how level of practice was then quantified (e.g. frequency versus duration of practice). Most importantly, there are very few studies of the effects of practice on outcome in MBCT, in particular in relation to risk of relapse to depression, the main outcome of interest in existing randomised controlled trials (Piet & Hougaard, 2011). Thus there is little scientific evidence to support the hypothesis that home practice is a key vehicle for change in MBCT or to guide clinicians when they are discussing home practice of mindfulness meditation with their participants.

A second issue concerns the fact that relatively little attention has been directed to the fact that even where an association between mindfulness practice and positive outcome is observed, the amount of practice a person chooses to engage in may be confounded with other factors. One obvious confounding variable is a participant's level of belief in, or preference for, their treatment: i.e. how logical it seems, how credible, and how much they feel it is likely to work in their particular case. Since the early days of evidence-based psychological treatment it has been repeatedly shown that a participant's belief in or preference for their treatment can have a significant predictive impact on subsequent outcome. For example, Iacoviello et al. (2007) found in a treatment trial for major depression that treatment preferences for psychotherapy versus pharmacotherapy affected subsequent therapeutic alliance, a known predictor of outcome in psychotherapy (e.g. Flückiger, Del Re, Wampold, Symonds, & Horvath, 2012). Likewise Kwan, Dimidjian, and Rizvi (2010) found in a trial of pharmacotherapy versus psychotherapy for depression that a mismatch between patient preference and allocated treatment was associated with increased attrition, reduced session attendance and poorer therapeutic alliance early in treatment, leading to indirect effects on patient outcome. In a final example from an early trial of Cognitive Therapy (CT), Fennell and Teasdale (1987) reported a correlation of *r* = −.76 between a Session 2 rating of plausibility of the treatment rationale of CT for depression and the final outcome, Montgomery-Asberg Depression Rating Scale depression score post-treatment. Associations between treatment preference or plausibility and subsequent outcome are not always straightforward (e.g. Steidtmann, Manber, Arnow, & Kocsis, 2012). However it has been suggested that treatment gains can be accounted for to a significant degree by early reductions in hopelessness that arise from the presentation of a plausible treatment rationale in the first sessions of treatment and the opportunity for the participant to explore the rationale as part of homework (e.g. Ilardi & Craighead, 1994). This has important implications. Like homework assignments in CT, meditation practice in MBCT is time consuming and challenging (often requiring individuals to expose themselves to previously avoided subjective experience, Segal et al., 2013). It is possible that strong preferences for a treatment and/or high perceptions of treatment plausibility may provide the motivation to adhere to home meditation practice, thus producing an association between treatment plausibility and engagement in home practice. Indeed, it may be that increased plausibility *itself* predicts outcome, and does so independently of home practice, through other mechanisms, such as those described above. It is therefore important to rule out the possibility that any relationship observed between amount of home practice and outcome is confounded with treatment plausibility.

Mindfulness practice can be quantified in a number of different ways, for example in terms of frequency and/or duration of longer formal meditation practices (for example, in MBCT, following a guided meditation CD, focussing on sensations in the body, sounds, thoughts and emotions), shorter formal practices (for example in MBCT, the 3-min breathing space), informal meditation practices (for example, in MBCT the cultivation of mindfulness in routine daily life activities), or both. There is no consensus in the broader meditation literature concerning whether frequency of practice or duration of practice is the most important variable for producing change or indeed whether formal or informal mindfulness practice is more important in this regard. Further, it is unclear whether a linear relationship would be expected to exist between amount of practice and observed benefits, or whether in fact once a threshold, or adequate minimum ‘dose’ of practice is reached, this is sufficient to produce change, at least in the context of relatively short and intensive MBIs. To examine these issues one recent study looked at the relationship between practice and outcome in participants receiving MBCT for bipolar disorder, considering both the cumulative number of sessions of formal meditation practice participants completed across treatment, and the comparison between those practicing formally for three or more days per week and those practicing less often (Perich, Manicavasagar, Mitchell, & Ball, 2013). Results showed there was a significant correlation between total number of days on which a formal meditation practice was undertaken and clinician rated depression at 12-month follow-up. Additionally, those who reported meditating on average three or more days per week had lower levels of depression and anxiety at 12-month follow-up than those reporting fewer days of formal practice. Likewise Hawley et al. (2014) analysed data from 32 participants attending either MBCT or Mindfulness Based Stress Reduction (MBSR) groups and identified an indirect association between amount of formal (but not informal) meditation practice and outcome (change in depressive symptoms on the Hamilton Rating Scale for Depression) which was partially mediated by reductions in rumination.

The primary purpose of the current study was to examine the association between home practice and outcome in 99 participants receiving MBCT as part of the Staying Well after Depression Trial (Williams et al., 2010, Williams et al., 2013). Following Perich et al., (2013) we examined (a) the relationship between the amount of formal home meditation practice completed over seven programme weeks, treated as a continuous variable, and hazard of relapse to major depression over follow-up; (b) the relative hazard of relapse for those engaging in a formal home practice on an average of three or more days per week (i.e. at least every other day/50% of recommended days) as compared to those engaging in formal home practice less often; and (c) the relationship between hazard of relapse to major depression and the total number of informal mindfulness practices completed over the treatment weeks. In each case we considered evidence for potential confounding of the relationship between home practice and outcome by treatment plausibility. It was hypothesised that:

a) There would be a significant association between average daily duration of formal home practice and hazard of relapse to depression.
b) Individuals who engaged in formal practice on at least 3 days per week would have a significantly lower hazard of relapse to depression than those who practiced less often,
c) That there would be a significant association between increased amount of informal home practice and reduced hazard of relapse to major depression
d) That increased ratings of treatment plausibility would be associated with greater formal and informal home practice, but
e) That the associations between formal and informal home practice would remain after adjusting for any significant confounding effect of treatment plausibility.

## Method

### Participants

**Fig. 1:**
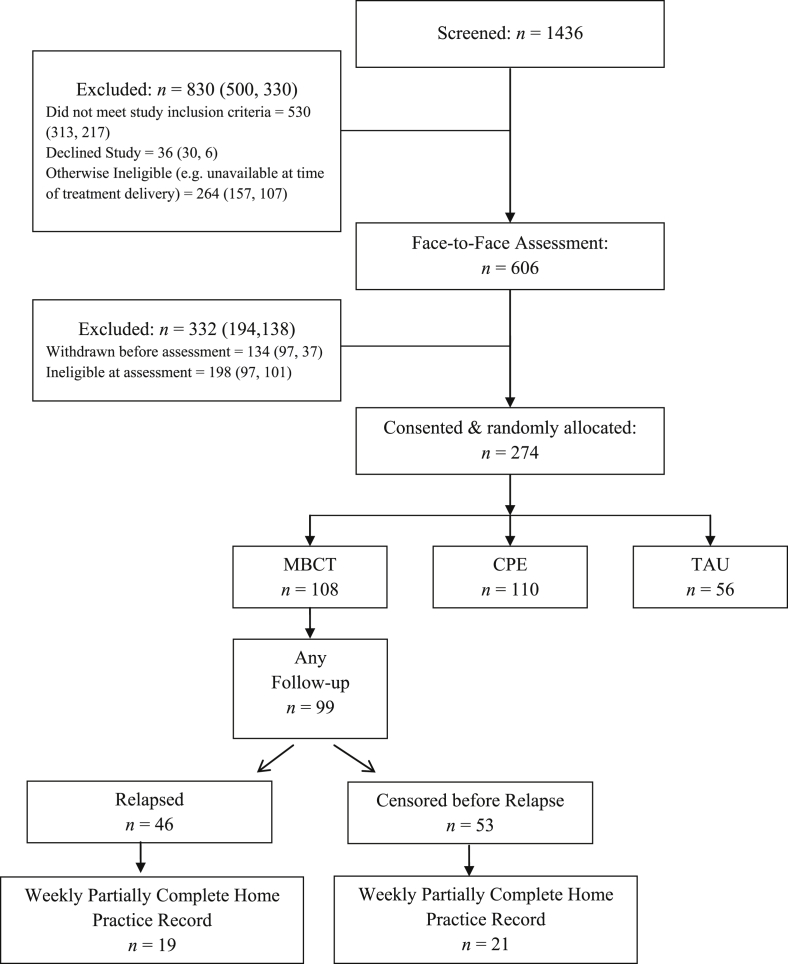
Participant flow through trial.

All participants in the trial had a history of at least three episodes of major depression and most (80%) had a history of suicidal ideation or suicidal behaviour. All were in remission on entry to the trial. Following previous randomised controlled trials of MBCT for relapse prevention in recurrent depression (Piet & Hougaard, 2011), the primary outcome of the study was hazard of relapse to a DSM-IV diagnosis of major depression over a 14 month follow-up period (from trial entry to 12 months post-treatment). In total 274 individuals were recruited to the trial of whom 108 participants were randomised to receive MBCT. Remaining participants were randomised to either an active psychological control treatment (cognitive psychological education, CPE) or to treatment as usual (TAU), with participants randomised in a ratio of 2:2:1 to MBCT, CPE, or TAU. Neither of these latter treatments included a home practice component and so data from these participants is not considered further. Of those participants randomised to receive MBCT, 99 provided follow-up data, enabling us to examine the association between home practice and subsequent outcome for this group (see Fig. 1 for participant flow). The mean duration of follow-up in participants receiving MBCT was 467 days from trial entry. Participants attended a median of 7 out of 8 treatment sessions with 89 participants (90%) attending 4 or more sessions of MBCT. In previous trials this has been regarded as an adequate minimum dose of treatment (Teasdale et al., 2000). Of those who provided follow-up data, 94% were Caucasian, 70% were female and participants had a mean age of 43.86 years (SD = 12.92). In total 44% were taking antidepressants at entry to treatment.

### Procedure

Participants were recruited from the community, from general practice and from mental health services in and around Oxford, England and Bangor, North Wales, from September 2008 until December 2011. Once recruited and having passed initial telephone screening for eligibility, participants attended the research clinic for a full assessment. We assessed eligibility through the Structured Clinical Interview for *DSM-IV*, the *Diagnostic and Statistical Manual of Mental Disorders* (SCID, First, Spitzer, Gibbon, & Williams, 2002) and gathered data on a range of clinical and cognitive variables. Inclusion criteria employed at baseline assessment were: (a) age between 18 and 70 years; (b) history of at least three episodes of Major Depression meeting DSM-IV-TR criteria (American Psychiatric Association, 2000), of which two must have occurred within the last five years, and one within the last two years; and (c) remission for the previous eight weeks. Potential trial participants were deemed *not* to be in recovery or remission, and hence *ineligible*, if they reported that at least one week during the previous 8 they had experienced *either* a core symptom of depression (depressed mood, anhedonia) *or* suicidal feelings plus at least one other symptom of depression. Episodes of depression must not have been attributable to bereavement, substances or a medical condition, and must have been of a severity to impair functioning. All participants provided informed consent prior to the baseline assessment and renewed consent prior to randomisation. Participants also required informed consent from their primary care physicians. The Oxfordshire Research Ethics Committee C and the North Wales Research Ethics Committee approved the trial in July 2008, and several subsequent operational changes. Thereafter an independent Trial Steering Committee and Data Monitoring Subcommittee oversaw the trial.

Exclusion criteria were: (a) a history of schizophrenia, schizo-affective disorder, bipolar disorder, current abuse of alcohol or other substances, organic mental disorder, pervasive developmental delay, primary diagnosis of obsessive-compulsive disorder or eating disorder, or regular non-suicidal self-injury; (b) positive continuing response to Cognitive Behaviour Therapy (CBT), (i.e. no relapse to depression since treatment with CBT, since CBT is known to reduce risk of relapse); (c) current psychotherapy or counselling more than once a month; (d) regular meditation practice (meditating more than once per month); or (e) inability to complete research assessments through difficulty with English, visual impairment, or cognitive difficulties.

All assessments were conducted using fully trained assessors. Following randomisation to MBCT, participants completed a pre-class interview with their MBCT instructor and attended 8 weekly group treatment sessions (see treatment section for further details below). Participants were reassessed for relapse to depression by an assessor blind to treatment allocation immediately after treatment and then 3, 6, 9 and 12 months after treatment. Full details of the procedure for this trial can be found in Williams et al., (2013).

### Measures

#### Recurrence of major depression

The primary outcome of the trial was time, in days, until relapse to major depression, defined as meeting relevant SCID criteria for major depression for at least two consecutive weeks since the previous assessment. Participants were asked to date the onset of their episode as accurately as possible. Where they could not give a precise date of onset we used an algorithm to approximate the date of onset for derivation of ‘a days to relapse' variable.^3^ We audio recorded all SCID assessments, and recruited two independent psychiatrists to reassess a sample of 91 follow-up interviews. Inter-rater reliability between the original assessor and an independent psychiatrist was 0.74, 95% CI [0.60, 0.87], with 87% agreement on whether relapse had occurred.

#### Home practice diary

All participants receiving MBCT were asked to practice on six days out of seven and to complete a daily home practice diary in which they recorded whether or not they had completed the assigned formal practice guided by CD, any scheduled shorter meditations (for example scheduled 3 min ‘breathing spaces’) and whether they had completed informal mindfulness practices (for example mindfulness of routine activities, unscheduled ‘additional breathing spaces’ initiated in response to stressful experiences, and ‘noticings’ – bringing mindful awareness to moments in daily life). Tick boxes were used by participants to record each element of home practice alongside a space to make any free response comments on their home practice for their own benefit and that of the class instructor. Home practice diaries were submitted to class instructors at the end of each week so that any difficulties with home practice could be addressed. For the purposes of the current study it was assumed that if a participant had made no entry to the practice diary on a given day (i.e. had not ticked any of the practice boxes), no home practice had been completed on that day (see later discussion of missing data). Two variables were computed to describe formal home practice: a) the mean duration of daily formal practice completed (in minutes) across the 7 weeks of treatment (no practice was recorded for week 8 as this was the final class); and b) following Perich et al. (2013) a binary variable specifying whether or not the participant had completed at least three long formal practices each week, on average (i.e. had completed home practice on at least 50% of recommended occasions). Because the duration of the main assigned formal home practice was very similar from day to day and week to week, we assigned each completed main formal practice an approximate duration of 40 min. Additionally, following Hawley et al. (2014), we included scheduled breathing spaces as an additional component of formal practice. These were assigned an approximate duration of 3 min, with a maximum of three recorded each day from weeks 3–7.

In addition to the formal practice variables we computed a variable to describe the approximate amount of informal mindfulness practice completed, focussing on mindfulness of routine activity (completed in weeks 1 and 2), unscheduled ‘additional’ breathing spaces (completed in weeks 4–7) and moments of mindfulness (weeks 1–7). It is much harder to determine exactly how long participants spent on informal mindfulness practices, since unlike the CDs for example, they had no fixed or specified duration. As a result we simply counted the number of ‘units’ of informal practice completed over the 7 weeks of recorded homework practice.

#### Treatment plausibility

Participants were asked to rate the plausibility of MBCT at the start of Session 2, on three scales from zero to ten. These scales assessed: (a) how sensible the treatment rationale seemed (from 0 = not at all logical, to 10 = extremely logical); (b) how confident the participant felt that the treatment would be successful (from 0 = not at all confident to 10 = extremely confident); and (c) the confidence that the participant would have in recommending the treatment to a friend with similar problems (from 0 = not at all confident to 10 = extremely confident). Treatment plausibility ratings were available for all three items for 88 of the 99 individuals providing follow-up data. Responses to the three items were highly consistent (Cronbach's alpha = .86), and therefore, after looking at the mean scores on each variable, the three items were summed to give a total treatment credibility score for subsequent analysis.

#### Hamilton Rating Scale for Depression (Hamilton, 1960)

We assessed residual depressive symptoms at trial entry by the Hamilton Rating Scale for Depression (HAMD, Hamilton, 1960), using the 17-item version of this interviewer-rated scale.

#### Childhood Trauma Questionnaire (Bernstein & Fink, 1997)

Early adversity and abuse were assessed using the Childhood Trauma Questionnaire (CTQ), an established self-report measure containing 28 items, validated against interview measures of childhood trauma and maltreatment (Bernstein and Fink, 1997, Bernstein et al., 1994).

### Treatment

#### Mindfulness-Based Cognitive Therapy (MBCT)

In this study MBCT comprised an individual pre-class interview followed by eight weekly two-hour classes including training in meditation skills such as sustained attentional focus on the body and breath, and adopting a decentred view of thoughts as passing mental events. The programme followed the original MBCT manual (Segal, Williams, & Teasdale, 2002), except for greater emphasis on patterns of thoughts and feelings that might be associated with suicidal planning, factors that maintain and exacerbate such patterns, and preparation of explicit action plans for suicidal crises. Participants were also invited to follow-up classes taking place six weeks and six months post treatment, respectively. Each follow-up class lasted for two hours and included meditation, discussion of discoveries and difficulties since the course ended, and how these were being dealt with by participants.

### Statistical analysis

The relationship between amount of home practice and hazard of relapse to major depression was analysed using Cox proportional hazards regression models (Cox, 1972, Singer and Willett, 2003). In each of these models the dependent variable was number of days to relapse or censoring (cases were censored at point of last follow-up) and the independent variable was number of minutes of formal home practice, informal home practice or number of days per week on which formal home practice was completed (<3 versus 3 or more). The treatment credibility variable was normally distributed. There was some evidence of non-normality for the variable quantifying total mean duration of daily formal practice (Kolmogorov–Smirnov = .093, *p* = .011), but there were no outlying values. Similarly there was evidence of non-normality (Kolmogorov–Smirnov = .116, *p* = .001), but no outlying values for the variable describing number of units of informal practice completed. Therefore for analyses examining associations between baseline variables (amount of practice with treatment credibility) we employed Spearman's non-parametric correlation coefficients.

### Missing data considerations

Using imputation to deal with problems of missing data in home practice records was not an option for the current analyses, because the issue of interest *was* the completion or non-completion of home practice. However it was not possible to distinguish with certainty between home practice records left blank because home practice had not been completed, and home practice records left blank because home practice had not been reported upon. We examined the number of participants who reported no home practice at all for a given week (i.e. submitted a completely blank sheet or did not submit a sheet). Seventy-five per cent of participants (*n* = 74) provided some home practice data on at least 5 weeks out of 7, indicating that whilst sporadic missing data was relatively common, more extensive missing data was relatively rare. We made a decision that in any instance where a home practice sheet item was left blank, or not returned, that it would be assumed that the unit(s) of practice to which it referred had not been completed. In cases where a participant had completed at least some elements of the home practice diary for a given week this assumption appeared reasonable, because it is clear that the participant had engaged, at least broadly, in the process of home practice reporting. However if a home practice sheet was not submitted at all, or was submitted completely blank, it was less clear whether the issue was with home practice completion, or reporting, or both. In order to check that any results identifying an association between amount of home practice and outcome were not simply an artefact of a broader disengagement with the programme, characterised by a failure to complete or submit home practice records, we therefore repeated the main analysis on the subsample of participants (*n* = 40) who submitted at least partially completed home practice sheet for every week of the course. The results of these analyses are presented in the results section that follows, alongside those for the whole participant group.

## Results

### Amount of practice completed

Participants reported completing the main formal meditation practice CD on an average of 3.36 days per week (*SD* = 1.77, range 0–6.43). Sixty-one participants reported following the main formal practice CD on three or more days per week on average, whilst 38 individuals followed the CD less frequently. The average duration of daily formal practice (main formal practice CD activity and scheduled breathing spaces) was 21.31 min (*SD* = 11.39). The mean number of units of informal practice (routine activity, noticing and informal breathing spaces) completed over the treatment weeks was 80.44 (*SD* = 53.37). The two variables quantifying amount of formal and amount of informal practice were highly correlated, *r*s (99) = .82, *p* < .001.

### Treatment plausibility ratings

On average participants gave a score of *M* = 7.29 (*SD* = 2.39) on a scale of 0–10 for the item asking how sensible the treatment seemed, a score of *M* = 6.22 (*SD* = 2.06) on a scale of 0–10 for confidence in treatment success and a score of *M* = 6.72 (*SD* = 2.36) on a scale of 0–10 for confidence in recommending the treatment to a friend. The combined mean treatment credibility score was *M* = 20.32 (*SD* = 6.12).

### Relationship between treatment plausibility and home practice

We explored the correlation between the combined rating of treatment plausibility (summed across the three items above) and amount of home practice. This indicated that treatment plausibility was not significantly correlated with mean daily amount of formal home practice, *r*s (88) = .10, *p* = .36, or informal home practice, *r*s (88) = .15, *p* = .17. There was no significant difference in treatment plausibility between individuals who practiced on less than three days per week and those who practiced more often, *t* (86) = .10, *p* = .92.

### Relationship between amount of formal home practice and outcome

Two Cox Proportional Hazard regression models were computed to test the hypothesis that amount of formal practice was associated hazard of relapse to major depression. The first model entered the continuous variable corresponding to the mean daily duration of formal home practice. There was a significant effect of amount of formal practice on hazard of relapse to major depression, *B* = −.03, *SE* = .013, Wald (1) = 5.51, *p* = .018, with a hazard ratio for relapse of *HR* = .97, *CI* = .947 to .995.

**Fig. 2:**
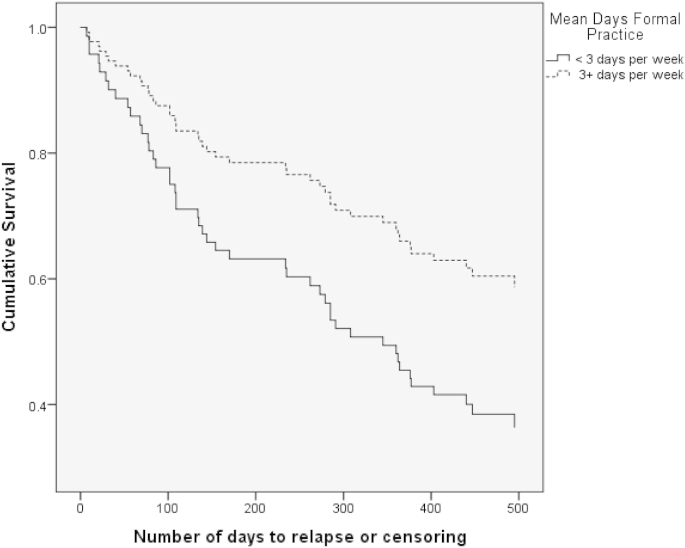
Hazard curve showing risk of relapse to depression over follow-up in participants practicing on three or more days per week on average, as compared to those practicing less frequently.

The second model considered a binary variable corresponding to <3 versus 3 + days of formal practice. When this variable was entered into the Cox regression analysis the model was again significant, *B* = −.64, *SE* = .30, Wald (1) = 4.66, *p* = .03, with a hazard ratio for relapse of *HR* = 0.53 *CI* = .30 to 0.94. Thus, those who practiced on three or more days per week were almost half as likely to relapse to major depression over the 12 month follow-up period as those who practiced on fewer than 3 days. Overall 39% of those who practiced on 3 or more days per week relapsed over 12 months follow-up in comparison to 58% of those who practiced on less than three days per week. Fig. 2 shows the hazard curves for participants falling into each home practice group.

### Sensitivity analysis

In order to examine possible bias associated with missing data we repeated the above analyses including only the *n* = 40 individuals who submitted at least partially completed home practice records for every week of the course. For the continuous variable of average daily minutes of formal home practice the results that were essentially the same, *B* = −.07 (*SE* = .03), Wald (1) = 5.10, *p* = .024, with a slightly reduced hazard ratio for relapse of *HR* = .93, but wider confidence intervals, *CI* = .88 to .99. For the dichotomised variable quantifying days of practice, the effect of practice on hazard of relapse was no longer significant, although given the lower power of this analysis the fact that it remained in a direction consistent with the overall findings: *B* = −1.10, *SE* = .76, Wald (1) = 2.11, *p* = .15, *HR* = .33, *CI* .08–1.47 is reassuring.

### Relationship between treatment plausibility and time to relapse to major depression

There was no significant relationship between treatment plausibility and hazard of relapse to major depression, *B* = −.01 (*SE* = .03), Wald (1) = .18, *p* = .67, HR = .99, *CI* = .94 to 1.04, indicating that in the current study treatment plausibility was unrelated to outcome.

The main outcomes of this trial, reported in Williams et al., (2013), indicated that residual symptoms of depression (HRSD) at entry to treatment, study centre and history of childhood trauma (CTQ) were all related to risk of relapse to depression in the sample as whole. We therefore re-ran the Cox Regression models relating formal practice to outcome including these additional variables, to determine whether the effect of practice on outcome was sustained after taking them into account. The relationship between average daily formal home practice and outcome remained significant, *B* = − .031 (*SE* = .012) Wald = 6.47, *p* = .01, *HR* = .97, *CI* = .95 to .99, as did the relationship between the binary formal practice variable and outcome, *B* = .80 (*SE* = .30) Wald = 6.95, *p* = .008, *HR* = .45, *CI* = .25 to .82. There were no significant differences between the two sites in amount of formal practice completed, *F* (1, 97) = .62, *p* = .43. There were also no significant correlations between amount of formal home practice and either residual symptoms of depression at baseline measured by the HRSD, *r*_s_ (99) = −15, *p* = .15, or levels of childhood trauma reported on the CTQ, *r*_s_ (99) = −.029, *p* = .78. Finally there was no significant effect of use of antidepressant medication at the baseline assessment on amount of either formal, *t* (97) = .70, *p* = .49 or informal, *t* (97) = .88, *p* = .38 home practice subsequently completed, and after entry of antidepressant usage at baseline assessment into the Cox Proportional Regression models the relationship between average minutes of daily practice, *B* = −.03 (*SE* = .013) Wald = 5.46, *p* = .02, *HR* = .97, *CI* = .95 to .995 and average days per week of meditation practice, *B* = −.66 (*SE* = .30) Wald = 4.67, *p* = .03, *HR* = .52, *CI* = .29 to .94 remained significant.

### Amount of informal home practice and outcome

A Cox Proportional Hazard regression model was computed to test the hypothesis that amount of informal practice was significantly associated with time to relapse to major depression. This indicated that there was no significant association between amount of informal practice and outcome, *B* = −.002 (*SE* = .002), Wald 1.74, *p* = .19, HR = 1.00, *CI* = .99 to 1.00. Despite the high degree of correlation between formal and informal practice, when both the continuous measure of formal practice and the measure of informal practice were entered together into a Cox Regression model, formal practice remained a significant predictor of time to relapse, *B* = −.059 (*SE* = .024), Wald (1) = 5.76, *p* = .014, *HR* = .94, *CI* = .90 to 99 and informal practice remained unrelated to time to relapse, *B* = .007 (*SE* = .005), Wald (1) = 2.015, *p* = .156, *HR* = 1.00, *CI* = 1.00 to 1.02.

## Discussion

Home meditation practice forms a central component of MBCT and it is hypothesised that through such practice class participants gain direct experience in relating differently to difficult thoughts, feelings and bodily sensations. The capacity to relate differently to such experiences is a key hypothesised relapse prevention mechanism in MBCT and thus a significant relationship between amount of home meditation practice and amount of benefit accrued from MBCT would be anticipated. However to date there have been relatively few studies to demonstrate the impact of practice on outcome, or to guide clinicians in their discussions of home practice with their participants. Further, no previous studies have examined the possibility that the amount of home practice completed by participants might be confounded with other factors which could themselves be related to treatment outcome, in particular perceptions of treatment plausibility.

The findings of this study confirm Hawley et al.'s (2014) and Perich et al.'s (2013) findings that formal home practice is related to outcome in MBCT, and indicates that people who engage in more formal home meditation practice have a significantly lower hazard of relapse to depression over 12 months follow-up. Indeed those who practiced on an average of three or more days per week were approximately half as likely to relapse to depression over 12 months follow-up as those who practiced less frequently. Furthermore, this study showed that differences in outcome were not accounted for by differences in early session ratings of treatment plausibility between those who went on to practice formally more frequently and those who practiced less. These findings confirm the importance of home practice as a factor that influences outcome of treatment with MBCT for recurrent major depression, and are consistent with the hypothesis that home practice supports learning in MBCT by providing participants with repeated experiences of responding to aversive thoughts, emotions and sensations in an ‘approach’ rather than ‘avoidant’ mode.

Like Hawley et al. (2014), we found no association between amount of *informal* home practice and outcome. It is possible that this relates to the greater difficulty in quantifying informal home practice. Whilst formal home practices (in particular, longer meditations guided by audio CD), have a prescribed duration and structure and are easier to capture through participant self-report, informal home practices (which encourage participants to generalise mindfulness to daily life activities and experiences), are by their nature less defined in terms of form or duration. In the current study informal home practice comprised participants' reported completion of unscheduled breathing spaces at times of stress, mindfulness of routine activities and ‘noticings’ (moments of mindfulness throughout the day). However these measures are likely to only crudely and partially capture the extent to which participants generalised their emerging mindfulness skills to daily life. In addition, it is possible that some informal mindfulness practices might actually increase in frequency in parallel to worsening mood as a person strives to reduce negative affect and stay well. A more in depth study of the use of informal practice in MBCT, the ways in which individuals generalise mindfulness to daily life, and the functions served by such activities are clearly important issues for future research, before it can be concluded that such practices do not contribute significantly to the effects of MBCT or other similar interventions on relapse to depression.

A limitation of the current study is that whilst we examined both average daily minutes of formal home practice and whether or not participants practiced on at least half the recommended days each week, we did not conduct more complex analyses to model either potential non-linear relationships between level of home practice and outcome, nor the effects of other thresholds for frequency or duration of home practice on subsequent hazard of relapse to depression. It would be interesting in future work to examine such effects in more detail in order to identify potential boundaries at which changes in level of home practice produce only minimal additional benefit. In view of this the findings concerning absolute reductions in hazard of relapse with increasing frequency of daily home practice should be interpreted cautiously. Indeed it is very likely that the relationship between level of practice and benefit accrued varies as a function of the characteristics and ‘starting point’ of a given individual. Clearly examining these issues requires further work, ideally in a larger cohort of patients incorporating both greater variability in home practice and a more refined characterisation of practice duration and frequency.

Contrary to our expectations we did not identify either a significant association between treatment plausibility and amount of home practice completed or a relationship between treatment plausibility and outcome. A previous pilot trial of MBCT for recurrent suicidal depression had shown that rates of attrition from treatment were quite high (30 per cent; Crane & Williams, 2010) and so in the current trial considerable effort was invested in early participant socialisation and treatment engagement, and in energetic follow-up of participants who were struggling or appeared to be at risk of drop out. One possibility is that these efforts resulted in the relatively high mean ratings of treatment plausibility observed, and have obscured associations that would otherwise have emerged between plausibility and outcome. Further research is required to explore what factors influence the extent to which individuals engage in and sustain home practice over time, particularly if our finding that home practice is important in reducing risk to relapse is replicated by others.

A further significant limitation of this study is that we did not gather similar data on continued home meditation practice after the completion of the eight week MBCT course. It is likely that participants who practice more during treatment might also be more likely to continue to practice after treatment, and that this might contribute significantly to home practice's prophylactic effects. However, in the absence of in depth data on meditation practice following the completion of treatment, we cannot know to what extent this was an important protective factor.

Another limitation is that we were not able to determine with certainty whether missing practice data reflected a person failing to practice, or failing to report upon their practice. In order to derive a conservative estimate of practice we assumed that where a practice diary was not completed on a given day or for a given week that no practice had taken place over that period. Thus, at the extremes a lack of practice may have been confounded with a broader disengagement with treatment. Reliance on self-report home practice data is a limitation shared with most previous studies, and it is reassuring that the results were almost identical when only those who provided at least some practice data on all seven homework records were analysed. However it would clearly be beneficial in future studies to explore the use of technology to map home practice more accurately (for example providing guided meditation audio files on a device that automatically logged time and date of access).

A final limitation is that we did not gather data on what exactly happened within sessions of formal home practice. Measuring the ‘quality’ or intentions of a meditation practice is clearly challenging, but it would certainly be of interest in future research to explore how participants engaged with their home practice in more detail in order to determine how important these factors are in moderating the effect of home practice on outcome.

Despite these limitations there are also significant strengths. Few studies have considered the effects of home practice on outcome in MBCT to date, and the current study was able to draw on a large and well characterised sample of participants to address this issue. Furthermore, we were able to rule out one obvious potential confounding variable, that a greater amount of home practice might be a consequence of increased treatment plausibility and influence outcome through this association. Our research suggests that clinicians can be confident in talking about the value of home practice as a means of reinforcing learning during MBCT. Future research is now required to refine our understanding of the mechanisms through which home practice reduces risk of relapse in depression and how informal meditation practices might be used to augment and enhance these effects.

## Conflict of interest

This study was funded by Wellcome Trust Grant GR067797, awarded to J. Mark G. Williams and Ian T, Russell (Trial Registration Number: ISRCTN97185214). The funders had no role in study design; in the collection, analysis and interpretation of data; in the writing of the report; and in the decision to submit the article for. All authors declare financial support for the submitted work from the Wellcome Trust; J. Mark G. Williams, Melanie J. V. Fennell, Thorsten Barnhofer, Sarah Silverton, Rebecca S. Crane, and/or their institutions have received honoraria or fees for lectures, workshops, courses, and/or educational presentations on mindfulness or mindfulness-based cognitive therapy (MBCT); J. Mark G. Williams, Melanie J. V. Fennell, Sarah Silverton, and Rebecca S. Crane have received royalties for books on mindfulness, including the MBCT manual (J. Mark G. Williams).

## Footnotes

3 When the participant gave a month of onset this was approximated to the 15th of the month. Where the participant stated that their episode began at the start of the month (e.g. ‘beginning of February’) onset was approximated to the 1st of the month. ‘Early’ in the month was approximated to the 8th and ‘late’ was approximated to the 22nd of the month. ‘End’ of the month was approximated to the final day of the month. Where these algorithms derived a duration of relapse of less than two weeks (for example the participant stated that the episode both started and ended in ‘January’ with no further details, leading to initial start and end dates of 15th January), the dates were expanded equally in both directions to produce a two week relapse period (e.g. 8th to 22nd) since the research assessor had always ensured that the symptoms had been present for at least two weeks.
